# Number of remaining teeth and health-related quality of life: the Korean National Health and Nutrition Examination Survey 2010–2012

**DOI:** 10.1186/s12955-019-1078-0

**Published:** 2019-01-09

**Authors:** Hyo-Eun Park, Hye Young Song, Kyungdo Han, Kyung-Hwan Cho, Yang-Hyun Kim

**Affiliations:** 1 Graduate School of Nursing, Korea University College of Nursing, Seoul, South Korea; 2 Department of Nursing, Suwon Women’s University, 72 Onjeong-ro, Gwonseon-gu, Suwon, Gyeonggi South Korea; 3 Graduate School of Nursing, Ewha Womans University of Korea, Seoul, South Korea; 4 Department of Medical Statistics, Catholic University College of Medicine, Seoul, South Korea; 5 Department of Family Medicine, Korea University College of Medicine, Korea University Anam Hospital, 73 Inchon-ro, Seongbuk-gu, Seoul, 02841 South Korea

**Keywords:** Remaining teeth, Quality of life, EQ-5D, Korean National Health and nutrition examination survey

## Abstract

**Objectives/aims:**

With the Euro-Qol-5 dimension (EQ-5D) system, we investigated the relationship between the number of remaining teeth and QoL using data from the Korean National Health and Nutrition Examination Survey (KNHANES), 2010–2012. A total of 17,417 participants, more than 19 years old, were finally included in this study (men = 7394 and women = 10,023). Through this study, we have discovered that the remaining teeth affect overall health and that the fewer number of them may indicate a lower quality of life, as well.

The quality of life according to the number of remaining teeth was assessed among Koreans using the Euro-Qol-5 dimension (EQ-5D) system.

**Method:**

The Euro-Qol-5 dimension (EQ-5D) system was used to measure the health-related QoL. Its five dimensions included mobility, self-care, usual activities, pain/discomfort, and anxiety/depression. The respondents were asked to choose one of the followings: G 1, no problems; G 2, some problems; and G 3, problematic, to best describe their health status for the five dimensions. Then, we assigned low QoL to G2 + G3 and high QoL to G1.

We used age, gender, economic income, educational level, residence, and marital status for the demographic variables and, drinking, smoking, exercise, BMI, and metabolic syndrome for health behaviors. Multiple logistic regression analysis was performed to examine the odds ratios (ORs) and confidence intervals (CIs) for the high QoL (G1) on the five categories of EQ-5D according to the number of remaining teeth. On the basis of the 0–15 remaining teeth group, we drew a comparison of the QoL between the 16–20 and 21–28 remaining teeth groups.

**Results:**

Subjects with 21–28 remaining teeth had higher QoL scores and had higher ORs of high QoL, especially for mobility (OR = 1.256, 95% CI = 1.056–1.495), self-care (OR = 1.441, 95% CI = 1.096–1.894), and usual activities (OR = 1.241, 95% CI = 1.022–1.508, respectively), than those with 0–15 remaining teeth after adjusting for age, sex, body mass index, smoking, drinking, exercise, income, education, and metabolic syndrome.

ORs from the high QoL had the tendency to increase as the number of remaining teeth increased (all p for trend < 0.05). However, there was no relationship between the number of remaining teeth and QoL in the pain/discomfort and anxiety/depression dimensions.

**Conclusion:**

The number of remaining teeth was associated with QoL, and subjects who had more teeth obtained higher QoL scores. The subjects in the high QoL group were especially associated with the components of EQ-5D such as mobility, self-care, and daily living.

## Background

QoL (Quality of life) scales provide both an objective measure of individual well-being and a self-reported measure of satisfaction [1]. Health-related QoL includes elements that may directly influence an individual’s physical, psychological, and social health, as well as their general well-being [2]. Wilson & Cleary defined it as subjective well-being associated with overall life satisfaction and happiness of individuals [3]. Various tools for measuring health-related QoL are also used to evaluate the general effects of healthcare policy on health status [4, 5]. Among these tools, the EuroQoL five-dimension questionnaire (EQ-5D) [6] has been widely used to measure health-related QoL in a simple and comprehensive manner [7]. The EQ-5D system consists of five sub-domains that measure mobility, self-care, usual activities, pain/discomfort, and anxiety/depression. Assessing the level of each domain helps evaluate the state of quality of life.

Many studies have evaluated the relationship between QoL and chronic diseases such as hypertension and diabetes. People with chronic diseases have lower QoL scores than those without them [8–11]. Some studies investigated the relationship between QoL and oral health status such as dry mouth, and also revealed an association between poor oral health status and low QoL [12, 13]. Moreover, there is a greater possibility that dental caries and periodontal diseases will lead to tooth loss. Which means masticatory and aesthetic functions will experience some accelerating declines. All this will place a limitation on their sociopsychological as well as social functions. Some studies indicated that tooth loss increases the individual’s sense of social alienation and isolation and as a result, exerts a negative influence on their QoL. [14–16]. Previous studies examined tooth loss and health-related QoL in the elderly and showed that there were inverse associations between them [17, 18, 19]. In addition, regarding the loss of teeth, those who experienced tooth loss and masticatory discomfort were reported to have lower health-related quality of life; the quality of life among the elderly seemed to be more affected than among those middle-aged [20, 21].

However, Fontanive et al. found no relationship between the number of remaining teeth and elements of health-related QoL [22]. Thus, the relationship between tooth loss and QoL remains unclear. Moreover, since most of the previous studies have targeted the elderly subjects, there have been insufficient studies designed for the whole adult population.

The EQ-5D is one of the most commonly used and of the simplest questionnaire tools to measure the overall health status as well as the quality of life. However, the validity and reliability of other QOL tools have yet to be established, so we have adopted the Korean version of EQ-5D for it has been verified by the National Health and Nutrition Examination Survey. With the qualified tool, we have investigated the health-related QoL and the number of remaining teeth in the Korean adults using data from the KNHANES (Korean National Health and Nutrition Examination Survey) during the period of 2010–2012.

## Methods

### Data collection and study population

The KNHANES used rolling samples and a stratified, multistage probability sampling design based on age, sex, and region in order to accurately represent the non-institutionalized civilian population of Korea. To ensure consistent and reliable performance and to reduce bias in the interviews and surveys, the KNHANES used a technical investigation team comprising of a nurse, a nutritionist, and a health science major, whose investigative performance had been regularly verified and maintained through training and field quality control.

The sampling framework of the Korean National Health and Nutrition Examination Survey was based on the most recent population census data available at the time of sample design. Representative samples were extracted from those who were more than one-year-old living in Korea. However, the data on population and housing census were outdated at the time of the design (2010–2012), so the resident population and apartment complex survey data were used as an extraction frame. For the sampling method, the two-stage stratification method was used with district and household as the first and second extraction units.

The 5th KNHANES consisted of 25,534 respondents and the study included participants aged 19 years or older; 5935 participants were under 19 years old and 2182 non-respondents were excluded. Finally, a total of 17,417 (men = 7394 and women = 10,023) valid participants completed the basic questionnaires, anthropometric and biochemical measurements.

The results from the sample data of the 5th KNHANES (2010–2012) were representative of the Korean population. To obtain unbiased estimation results, we used a composite sample analysis module that took into account the weighting, stratification, and cluster variables. Weights were calculated using design weights reflecting the sampling rate, non-response adjustment, post-stratification, and extreme weighting. For the stratification variable, we used a variance estimation layer that integrated some sample design layers. For the colony variables, the first extraction unit was used.

The KNHANES received annual deliberation and approval from the institutional review board of the Korea Centers for Disease Control and Prevention (2010-02CON-21-C 2011-02CON-06-C 2012-01EXP-01-2C). All participants gave their written consent.

### Sociodemographic variables and general health behavior

Trained interviewers recorded sociodemographic and lifestyle variables for the participants. Education level was categorized into two groups, high school graduates or higher (education ≥13 years). Household income was calculated by standardizing monthly income by the number of family members (monthly income/number of family members). a.

The average monthly household income was divided by 4 quartiles. And the income was classified as follows: ‘Under 1 million won (US 928.1$)’, ‘Over 1 million won ~ 2 million won (US 18,57.01$)’, ‘Over 2 million won ~ 3 million won (US 2785.52$)’, and ‘over 3 million won’. For your information, Korea’s minimum wage has now been 7530 won per hour (US 6.72/1 h).

The residence type was divided into two, rural or urban areas. Alcohol consumption was categorized into two groups in accordance with their answers on the self-report questionnaire: monthly alcohol use or not within the last year [23]. For smoking, the subjects were categorized into two groups in accordance with their answers on the self-administered questionnaire: current smoker or not. Exercise status was based on responses to a modified form of the International Physical Activity Questionnaire for Koreans, with individuals regarded as regular physical exercisers if they performed moderate exercises more than five times a week, over 30 min per session, or performed vigorous exercises more than three times a week, over 20 min per session [24].

### Anthropometric measurements

We measured waist circumference to the nearest 0.1 cm on a horizontal plane at the midpoint between the costal margin and the iliac crest at the end of normal expiration. Height and weight were measured to the nearest 0.1 cm and 0.1 kg, respectively, with the subject wearing lightweight clothing. The body mass index (BMI) was calculated by dividing weight in kilograms by height in square meters (kg/m^2^). We measured systolic and diastolic blood pressure (BP) three times using a standard mercury sphygmomanometer (Baumanometer; W. A. Baum Co., Inc., Copiague, NY, USA). Blood pressure was measured twice at five-minute intervals and its measurements were averaged for analysis. Blood pressure was measured three times, in total, and the secondary and tertiary mean blood pressures were used as final systolic and diastolic blood pressure.

### Biochemical measurements

After a minimum of 8 hours of fasting, blood samples were obtained. The serum enzyme levels of TC, TG, LDL-C, and HDL-C in the blood samples were measured using a Hitachi Automatic Analyzer 7600 (Hitachi, Tokyo, Japan) after the samples were delivered to the Central Testing Institute in Seoul, Korea. The LDL-C level was calculated using the Friedewald’s formula in subjects with a TG level ≤ 400 mg/dL and was measured using commercially available kits (Cholestest® LDL; Sekisui Medical, Tokyo, Japan) in subjects with a TG level > 400 mg/dL. The fasting blood glucose level was measured after they fasted for 8 hours or more. In addition to fasting blood glucose, the hemoglobin A1c test was also performed. Fasting blood glucose was measured with Tosoh G8 (Tosoh/Japan) using the high-performance liquid chromatography method and with HLC-723G8 HbA1C kit.

### The number of remaining teeth

In the 5th KNHANES, the applicants visited the mobile examination center; dentists collected the information on the applicants’ teeth condition and on the distribution of their teeth while examining their oral cavities.

Since July 2007, the Center for Disease Control and Prevention (KCDC) established the ‘Specialized Survey Team’ in order to carry out a stable investigation with the introduction of the survey system throughout the year. The Specialized Survey Team consisted of specialists/researchers; nurses, nutritionists, dentists, and health science students.

Meanwhile, in counting the total number of remaining teeth, the third molars were excluded. The participants were then classified into one of the three groups based on the number of remaining teeth: ≤15, 16–20, or 21–28 [25, 26]. For your reference, the average number of teeth of an individual is 28 except for some wisdom teeth. Of them, molar teeth play a key role in chewing and consuming food. Under the situation, if you have 8 M pulled out, 4 from maxilla (upper jaw) and 4 from mandible (lower jaw), you will finally have 20 teeth of 28 in total. This means, given that food consumption fills a large part of your life, you may miss out on a big opportunity to lead a happier life in a way. Thus, this is partly why most previous studies have classified teeth groups according to the number of molars.

And, as mentioned earlier, on the basis of the 0–15 remaining teeth group, we drew a comparison of the QoL between the 16–20 and 21–28 remaining teeth groups.

As part of quality control, training was provided by the examiners. This is to minimize measurement errors in the number of remaining teeth.

### Measuring QoL

To examine health-related QoL, this study utilized the EQ-5D system, which was developed by the EuroQoL group for clinical and economic evaluation [6, 27, 28]. The EQ-5D is divided into five dimensions. They include mobility, self-care, usual activities, pain/discomfort, and anxiety/depression. The respondents were asked to choose one of the followings: G 1, no problems; G 2, some problems; and G 3, problematic, to best describe their health status for the five dimensions. Then, we assigned low QoL to G2 + G3 and high QoL to G1. This study calculated the total score and five categories in different method. According to the weight of Center for Disease Control and Prevention in Korea, the five items of EQ-5D index are converted to 15 points (highest score) making the total score 1. In table3, instead of adding up the Euro-Qol 5 score, we used each score of 5 categories of Euro-Qol 5.

### Metabolic syndrome

The presence of metabolic syndrome is determined by the guidelines of the American Heart Association/National Heart, Lung, and Blood Institute Scientific Statement Criteria for Asians [29]. To be diagnosed as having metabolic syndrome, three or more of the following criteria must be fulfilled: (1) waist circumference ≥ 90 cm in men and ≥ 80 cm in women; (2) fasting triglycerides ≥150 mg/dL or the use of lipid-lowering medication; (3) high-density lipoprotein cholesterol < 40 mg/dL in men and < 50 mg/dL in women or the use of medication; (4) blood pressure ≥ 130/85 mmHg or the use of antihypertensive agents; and (5) fasting blood glucose ≥100 mg/dL or the current use of antidiabetic drugs.

### Statistical analysis

Complex sample analysis was conducted using SAS version 9.3 (SAS Institute Inc., Cary, NC, USA). SAS survey procedures were used to reflect the complex sampling design, and the sampling weights for the KNHANES were applied to provide nationally representative prevalence estimates. For the general characteristics of the sample population, statistical differences in means ± standard errors (SEs) for continuous variables and percentages (SEs) for categorical variables were identified using one-way ANOVAs and chi-square tests, respectively. ANOVA was performed to identify differences in the number of teeth in relation to the three levels of self-reported health status (G1, G2, and G3) in the EQ-5D index. Chi-square tests were used to determine whether the number of teeth affected the percentage of respondents who reported low QoL (G2 + G3). T-tests and Chi-square tests were conducted to assess the relationship between the number of teeth and the presence of metabolic syndrome and having a low QoL. Multiple logistic regression analysis was performed to examine the odds ratios (ORs) and confidence intervals (CIs) for the high QoL (G1) on the five categories of EQ-5D according to the number of remaining teeth. The adjusted covariates were age, sex, BMI, smoking, drinking, exercise, income, education, and metabolic syndrome. And we performed linear trend test. *P* values less than 0.05 were considered statistically significant and all statistics were conducted using two-tailed tests.

## Results

Table 1 shows the general characteristics of the three groups according to the number of remaining teeth. The 5th KNHANES included a total of 25,534 respondents. To be more specific, the study included participants aged 19 or older; 5935 participants were under 19 years old and 2182 non-respondents were excluded.

**Table 1:** Descriptive statistics for the sample according to the number of remaining teeth

Variables	Number of remaining teeth			*p*
	0–15	16–20	21–28	
Unweighted n	2169	1068	7186	
Age (years)	69.6 ± 0.31	62.3 ± 0.51	42.1 ± 0.19	< 0.001
Men (yes, %)	43.8 (1.3)	47.4 (1.9)	50.0 (0.4)	< 0.001
BMI (kg/m^2^)	23.5 ± 0.09	24.3 ± 0.13	23.7 ± 0.04	< 0.001
Waist circumference (cm)	83.1 ± 0.28	84.6 ± 0.35	80.7 ± 0.14	< 0.001
Current smoking (yes, %)	21.1 (1.2)	19.8 (1.5)	24.2 (0.5)	0.004
Alcohol intake (yes, %)	37.2 (1.3)	45.9 (1.8)	61.1 (0.6)	< 0.001
Regular exercise (yes, %)	14.3 (1.1)	19.1 (1.5)	20.1 (0.5)	< 0.001
Income (Q1) (yes, %)	48.6 (1.5)	34.3 (1.9)	11.8 (0.5)	< 0.001
Education ≥13 years (yes, %)	19.1 (1.2)	30.2 (1.9)	78.3 (0.6)	< 0.001
Urban living (yes, %)	60.7 (2.9)	69.8 (2.9)	82.6 (1.6)	< 0.001
Having a spouse (yes, %)	65.6 (1.3)	77.8 (1.7)	81.8 (0.8)	< 0.001
Metabolic syndrome (yes, %)	48.9 (1.5)	49.8 (2.0)	22.8 (0.4)	< 0.001
EQ-5D	0.850 ± 0.005	0.897 ± 0.006	0.960 ± 0.001	< 0.001

Data presented as mean ± SE or percentage (SE)

The *p*-values were obtained by one-way analysis of variance or chi-square test

EQ-5D: The EuroQoL five dimension questionnaire (Mobility, Self-care, Usual activities, Pain/discomfort, Anxiety/depression)

Subjects with 0–15 remaining teeth were the oldest (69.6 years) and had the lowest BMI (23.5 kg/m^2^). Also, they had the lowest proportion of men, alcohol intake, regular exercise, education ≥13 years, urban living, and having a spouse (all *p* < 0.001), but the proportion of the lowest income (Q1) was the highest. Subjects with 21–28 remaining teeth were the youngest (42.07 years) and had the highest proportion of men, current smoking, alcohol intake, regular exercise, education ≥13 years, urban living, and having a spouse (all *p* < 0.005), but they had the lowest WC and proportion of income (Q1) and metabolic syndrome (all *p* < 0.001). The EQ-5D score was the lowest in the subjects with 0–15 remaining teeth (0.850 ± 0.005), followed by the subjects with 16–20 remaining teeth (0.897 ± 0.006), and the highest in the subjects with 21–28 remaining teeth (0.960 ± 0.001) (*p* < 0.001).

Table 2 shows the mean number of remaining teeth according to the five dimensions of EQ-5D divided into three levels (G1 to G3). In all five dimensions, G1 had the highest number of remaining teeth and G3, the lowest number of them (all *p* < 0.001).

**Table 2:** The average number of teeth according to EQ-5D^a^

5 categories of EQ-5D^a^	Number of remaining teeth	*p*
Mobility		< 0.001
G1. I have no problems walking about	25.51 ± 0.06	
G2. I have some problems walking about	19.66 ± 0.22	
G3. I am confined to bed	14.71 ± 1.21	
Self-care		< 0.001
G1. I have no problem with self-care	25.03 ± 0.07	
G2. I have some problem with washing or dressing myself	17.47 ± 0.49	
G3. I am unable to wash or dress myself	13.18 ± 1.59	
Usual activities		< 0.001
G1. I have no problem performing my usual activities	25.24 ± 0.06	
G2. I have some problem performing my usual activities	19.46 ± 0.28	
G3. I am unable to perform my usual activities	15.47 ± 0.85	
Pain/discomfort		< 0.001
G1. I have no pain or discomfort	25.29 ± 0.07	
G2. I have moderate pain or discomfort	23.26 ± 0.15	
G3. I have extreme pain or discomfort	17.70 ± 0.52	
Anxiety/depression		< 0.001
G1. I am not anxious or depressed	24.92 ± 0.07	
G2. I am moderately anxious or depressed	23.60 ± 0.18	
G3. I am extremely anxious or depressed	20.40 ± 0.97	

Data presented as mean ± SE

The *p*-values were obtained by analysis of variance

^a^EQ-5D: The EuroQoL five dimension questionnaire

The groups divided by the number of remaining teeth were classified into those with high QoL (G1) and with low QoL (G2 + G3), and the odds ratios of the groups were compared in accordance with the five dimensions of the EQ-5D. This study calculated the total score and five categories in different method. According to the weight of Center for Disease Control and Prevention in Korea, the five items of EQ-5D index are converted to 15 points (highest score) making the total score 1. In logistic regression, it used each score of five areas of EURO-Qol instead of using EURO-Qol five total score.

Table 3 describes the details. There was a significant tendency to increase in the mean EQ-5D score as the number of remaining teeth increased (p for trend < 0.001). The OR for high QoL was more than 1 at mobility, self-care, and usual activities in the subjects with 21–28 remaining teeth (OR; 95% CI = 1.256;1.056–1.495, 1.441;1.096–1.894, and 1.241;1.022–1.508, respectively). These values tended to increase as the number of remaining teeth increased (all p for trend < 0.05).

**Table 3:** Odd ratios for the number of remaining teeth to each component of the EQ-5D

Number of teeth	Odds ratio (95% CI)^b^					
	EQ-5D^a^ Total score^c^	Mobility	Self-care	Usual activity	Pain/discomfort	Anxiety/depression
0–15	0.912 ± 0.007	1	1	1	1	1
16–20	0.934 ± 0.007	1.150	1.238	1.139	1.133	0.915
		(0.914–1.446)	(0.914–1.446)	(0.877–1.480)	(0.902–1.424)	(0.685–1.223)
21–28	0.944 ± 0.004	1.256	1.441	1.241	1.160	0.851
		(1.056–1.495)	(1.056–1.495)	(1.022–1.508)	(0.990–1.360)	(0.679–1.066)
*P for trend*	< 0.001	0.011	0.009	0.029	0.073	0.164

The *p*-values were obtained by multiple logistic regression analysis

^a^defined as G1 and reference group (G2 + G3) (Grade 1: no problems; Grade 2: some problems; Grade 3: severe problem),

^b^Adjusted for age, sex, body mass index, smoking, drinking, exercise, income, education, and metabolic syndrome

^c^EQ-5D index was calculated according to the weight of Center for Disease Control and Prevention in Korea. The total of index five items is converted to 15 points (highest score) making the total score 1

EQ-5D; The EuroQoL five-dimension questionnaire, and CI; confidence interval

Regarding the dimension of mobility(LQ-1EQL), it has been discovered that groups with more teeth had the linear trend of the increasing odds ratios for mobility QoL(p for Trend = 0.011). Also, compared to the group with 0~15 remaining teeth, the group with 21~28 experienced 1.256 times higher odds ratios for mobility QoL(LQ-1EQL) (95% CI: 1.056–1.495).

In a similar vein, for the dimension of self-care (LQ-2EQL), it has been found that groups with more teeth had the linear trend of the increasing odds ratios for self-care QoL (p for Trend = 0.009). Also, compared to the group with 0~15 remaining teeth, the group with 21~18 experienced 1.441 times higher odds ratios for self-care QoL (LQ-2EQL) (95% CI: 1.096–1.894).

With regard to the dimension of usual activities (LQ-3EQL), groups with more teeth had the linear trend of the increasing odds ratios for usual activities QoL (p for Trend = 0.029). Likewise, compared to the group with 0~15 remaining teeth, the group with remaining 21~18 experienced 1.241 times higher odds ratios for usual activities QoL (95% CI: 1.022–1.508).

However, there was no correlation between the number of remaining teeth and two categories of EQ-5D (pain/discomfort and anxiety/depression).

## Discussion

This study has indicated an association between the number of remaining teeth and health-related QoL assessed through the EQ-5D index. Subjects with 21–28 remaining teeth had higher QoL scores and had higher ORs of high QoL, especially for mobility, self-care, and usual activities than subjects with 0–15 remaining teeth after adjusting for all covariates [12, 18, 30, 31]. Thus far, many studies have investigated the relations of tooth loss and HRQoL based on self-reported tooth loss and the presence of metabolic syndrome. Against the background, in this study, the subjects had a checkup for their teeth by dentists. That is, this study analyzed the number and status of remaining teeth using data that was not based on self-reports but was obtained through examination by trained dentists. Therefore, it is expected to have as accurate results as it can.

Currently, the Short Form-36 Health Survey [32], Quality of Well-Being Scale [33], Health Utility Index [34, 35], World Health Organization QoL-BREF [36], and EQ-5D are being used in the assessment of QoL. The EQ-5D has been one of the simplest and of the most widely accepted tools to measure the overall health status as well as the quality of life, especially after translated into various languages and adapted to different culture and different countries, and after a unique weighted value was developed [37]. Moreover, it is considered to be one of the best tools for predicting quality-adjusted life years, used in health surveys of population groups, and for the clinical and economical evaluation of healthcare services [38, 39].

Brennan et al. reported that oral health affects general QoL [40–42] and this association has also been found in several studies [30, 43, 44] although they have focused only on the elderly population. Individuals with poor oral health are more likely to have trouble eating food and receiving adequate nutrition. Furthermore, oral pain, chewing difficulties, speech problems, and limited mobility of the jaws affect not only activities of daily living (ADL), but also interpersonal relationships [45, 26, 46]. According to Locker et al., oral disabilities affect daily life and further, may result in serious dysfunctions in many areas as individuals get older [47]. Tooth loss is one of the most severe damage to oral health status. Further, it can exert a highly adverse effect on aesthetic and functional areas of oral health. And, various factors play a crucial role in the loss of teeth; Of them, biological and socioeconomic factors fill a large and significant part of tooth loss-related oral health [46]. Generally, individuals’ state of health is strongly affected by their socioeconomic status. And as with all diseases, socioeconomically disadvantaged groups will be more at risk than others and will be placed under unfavorable conditions in health care, as well. The same goes for oral health. As constantly mentioned in this article, differences in the number of teeth can lead to different HRQoL; The more teeth they have, the higher their QoL will be and the higher income they have, the more teeth they have [12, 13, 17, 18]. In other words, individuals on higher incomes will invest more time and money in their proper and enough dental care. And as they have more teeth, they can enjoy a more variety of food. Given that food intake fills a large part of their life, those with more teeth would live life at a higher level. This will be mentioned later once again.

Han and Yom [31] and Miura et al. [48] found a correlation between oral health and ADL in the elderly population living in rural areas and Catović et al. [49] discovered that old people who had trouble engaging in ADL were more likely to have poorer oral health. These results propose that oral health is associated with activities bidirectionally. However, a study by Naito et al. [50] found that oral health did not affect ADL of senior citizens in nursing facilities and Fontanive et al. [22] did not find any associations between the number of remaining teeth and components of QoL. Under the circumstances, more studies are needed to build up a stronger relationship between oral health and QoL, especially in elderly populations.

The mechanism in the relationship between the number of remaining teeth and QoL can be explained by morbidity. In this study, subjects with metabolic syndrome had fewer teeth than those without it. Research conducted in Korea and Finland has pointed out that chronic diseases like diabetes are associated with tooth loss and other dental issues, leading to a lower QoL, [51, 52] based on the assumption from the studies that metabolic syndrome affects the relationship between the QoL and remaining teeth. Metabolic syndrome was set as a control variable in multiple logistic regression analysis.

The number of remaining teeth and low QoL with or without metabolic syndrome are shown in the Appendix table. Subjects with metabolic syndrome had a fewer number of teeth compared to those who did not have it (22.8 vs. 25.7, respectively) and they showed a higher proportion of the low QoL at all five dimensions of EQ-5D than subjects without metabolic syndrome (all *p* < .001).

Loesche [53] and Buhlin et al. [54] also found that arteriosclerosis reduces the blood and oxygen supply available to periodontal tissues, leading to periodontal disease and, in severe cases, to tooth loss. Moreover, limitations on consuming some types of food and reduction in the amount of food may cause tooth loss by undernourishment. Chewing Difficulty on food could also have a direct impact on QoL by eliminating the happiness derived from the consumption of a meal, especially for the elderly [55, 56]. Furthermore, tooth loss could also influence pronunciation and aesthetics, which in turn could promote a sense of social isolation as individuals choose to avoid normal social activities and interpersonal relationships [12, 57, 58].

Inversely, some studies showed that replacing a patient’s missing tooth with an artificial one improves their QoL. The use of a full set of dentures enhances the ability to chew and improves health-related QoL [59–61]. Therefore, given these, we postulated that there were bidirectional relationships between the number of remaining teeth and QoL.

This study, however, did not find a significant association between the number of remaining teeth and pain/discomfort or anxiety/depression. In fact, most previous studies [31, 48] pointed out that the oral status and the quality of emotional life of the elderly were associated with pain, anxiety, and depression. The differences between aforementioned studies and the present study may be a result of the different inclusion criteria such as age, some specific diseases, and the sample size, so precisely designed studies are needed for this issue in the future.

Strictly speaking, this study has some limitations. First, this is a cross-sectional-designed study. Therefore, it is difficult to establish a causal relationship between the number of remaining teeth and QoL. Second, difficulty in chewing is closely linked to the lack of opposing pairs in the upper and lower jaws, so future research should also examine whether the subjects have opposing pairs of teeth. Third, for we only included Korean adults, the result of this study is hard to be generalized to other ethnicities. Fourth, the data are from the 5th edition of the survey, so they are a little old. That means we could not include the recent trend. Fifth, the Qol is greatly influenced not only by the number of remaining teeth but by the distribution of lost teeth. However, we could not put the information on the distribution of lost teeth in our study.

Despite these limitations, this study has some strengths. It is the first study to verify the association between the number of remaining teeth and health-related QoL of representative Korean adults after controlling for a number of potentially confounding variables. This study also analyzed the number of remaining teeth using data that was not based on self-assessment but was collected directly by dentists during a clinical dental examination of their patients.

## Conclusion

The number of remaining teeth was associated with QoL, and subjects who had more teeth obtained higher QoL scores. High QoL was especially associated with the components of EQ-5D such as mobility, self-care, and daily living. The group of more remaining teeth showed a high quality of life especially in relation to activity. Thus, it was proven that the more the remaining teeth, the higher the quality of life because there were more movements.

Further studies are needed to prove the causal relationship between the number of remaining teeth and QoL, while also considering the number of opposing pairs and the presence of artificial teeth, such as implants. In addition, it is necessary to investigate the correlation between the loss of tooth position and quality of life. Last but not least, we suggest that we study the physiological mechanism of tooth loss in detail by examining the relationship between metabolic syndrome and remaining teeth.

## Appendix

**Table 4:** The number of remaining teeth and the proportion of low quality of life^a^ with or without metabolic syndrome

Metabolic syndrome	Number of teeth	EQ-5D components				
		Mobility	Self-care	Usual activities	Pain/discomfort	Anxiety/depression
Yes	22.8 ± 0.1	22.4 (0.7)	6.4 (0.4)	13.8 (0.6)	27.9 (0.9)	13.0 (0.6)
No	25.7 ± 0.1	8.0 (0.3)	2.0 (0.2)	4.8 (0.3)	17.3 (0.5)	9.2 (0.3)
p	< 0.001	< 0.001	< 0.001	< 0.001	< 0.001	< 0.001

Data presented as mean ± SE or percentage (SE)

The *p*-values were obtained by t-test or chi-square test

^a^defined as G2 + G3 (Grade 1: no problems; Grade 2: some problems; Grade 3: severe problem)

EQ-5D; The EuroQoL five-dimension questionnaire

**Table 5:** Odd ratios for the general characteristics to each component of the EQ-5D

Odds ratio (95% CI)					
	Mobility	Self-care	Usual activity	Pain/discomfort	Anxiety/depression
Age	0.957 (0.949,0.965)	0.941 (0.93,0.953)	0.957 (0.949,0.965)	0.982 (0.977,0.987)	0.991 (0.984,0.997)
Men	0.595 (0.512,0.692)	0.839 (0.648,1.087)	0.701 (0.579,0.849)	0.582 (0.518,0.654)	0.428 (0.361,0.509)
BMI	0.936 (0.915,0.958)	0.989 (0.957,1.022)	0.984 (0.96,1.008)	0.975 (0.959,0.992)	0.997 (0.975,1.019)
smoking	0.708 (0.579,0.865)	0.622 (0.458,0.843)	0.701 (0.554,0.887)	0.86 (0.74,1)	0.746 (0.61,0.911)
Alcohol	1.315 (1.131,1.528)	1.261 (1,1.59)	1.339 (1.14,1.574)	1.147 (1.028,1.279)	1.109 (0.957,1.284)
exercise	1.065 (0.909,1.247)	1.126 (0.835,1.519)	0.958 (0.793,1.159)	0.961 (0.847,1.091)	0.969 (0.823,1.142)
Income (Q1)	0.548 (0.474,0.634)	0.521 (0.397,0.684)	0.412 (0.348,0.488)	0.616 (0.545,0.695)	0.495 (0.418,0.587)
Education ≥13 years	2.464 (2.078,2.922)	1.709 (1.269,2.303)	2.169 (1.772,2.655)	1.548 (1.359,1.762)	1.272 (1.063,1.523)
spouse	0.838 (0.729,0.964)	0.734 (0.587,0.917)	0.734 (0.624,0.862)	0.894 (0.791,1.01)	0.91 (0.775,1.069)

The *p*-values were obtained by multiple logistic regression analysis

Adjusted for age, sex, body mass index, smoking, drinking, exercise, income, education, and metabolic syndrome

EQ-5D; The EuroQoL five-dimension questionnaire

## Abbreviations

ADL: Activities of daily living
BMI: Body Mass Index
EQ-5D: Euro-Qol-5 dimension
KNHANES: Korean National Health and Nutrition Examination Survey
QoL: Quality of life
WC: Waist circumference

## References

[1] Korea National Institute of Special Education. The dictionary of special education. Seoul: How; 2009.

[2] Spilker B, Revicki DA, Spilker B (1996). Taxonomy of quality of life. Quality of life and Pharmacoeconomics in clinical trials.

[3] Wilson IB, Cleary PD (1995). Linking clinical variables with health-related quality of life: a conceptual model of patient outcomes. JAMA.

[4] Burström K, Johannesson M, Diderichsen F (2001). Swedish population health-related quality of life results using the EQ-5D. Qual Life Res.

[5] Wallander JL, Varni JW (1998). Effects of pediatric chronic physical disorders on child and family adjustment. J Child Psychol Psychiatry.

[6] The EuroQoL Group (1990). EuroQoL-a new facility for the measurement of health-related quality of life. Health Pol.

[7] Han MA, Ryu SY, Park J, Kang JG, Kim KS (2008). Health-related quality of life assessment by the EuroQoL-5D in some rural adults. J Prev Med Public Health.

[8] Rubin RR, Peyrot M, Rubin RR, Peyrot M (1999). Quality of life and diabetes. Diabetes/Metab Res Rev.

[9] Glasgow RE, Ruggiero L, Eakin EG, Dryfoos J, Chobanian L (1997). Quality of life and associated characteristics in a large national sample of adults with diabetes. Diabetes Care.

[10] Zygmuntowicz M, Owczarek A, Elibol A, Chudek J (2012). Comorbidities and the quality of life in hypertensive patients. Pol Arch Intern Med.

[11] Willson IB, Cleary PD (1995). Linking clinical variables with health-related quality of life. JAMA.

[12] Kim SH, Lim SA, Park SJ, Kim DK (2004). Assessment Oral health-related quality of life using the Oral health impact profile (OHIP). J Korean Acad Oral Health.

[13] Ikebe K, Matsuda K-i, Morii K, Wada M, Hazeyama T, Nokubi T (2007). Impact of dry mouth and hyposalivation on oral health-related quality of life of elderly Japanese. Oral Surg, Oral Med, Oral Pathol, Oral Radiol, Endod.

[14] Somsak K, Kaewplung O. The effects of the number of natural teeth and posterior occluding pairs on the oral health-related quality of life in elderly dental patients. Gerodontology 2014 March 6 [Epub]. doi: 10.1111/ger.12112. 10.1111/ger.12112 24597864

[15] Lawrence HP, Thomson WM, Broadbent JM, Poulton R (2008). Oral health-related quality of life in a birth cohort of 32-year olds. Community Dent Oral Epidemiol.

[16] Kim SH, Lim SA, Park SJ, Kim DK (2004). Assessment of Oral health-related quality of life using the Oral health impact profile (OHIP). J Korea Acad Oral Health.

[17] Steele JG, Sanders AE, Slade GD, Allen PF, Lahti S, Nuttall N (2004). How do age and tooth loss affect oral health impacts and quality of life? A study comparing two national samples. Community Dent Epidemiol.

[18] Chen Y-F, Yang Y-H, Chen J-H, Lee H-E, Lin Y-C, Ebinger J (2012). The impact of complete dentures on the oral health-related quality of life among the elderly. J Dent Sci.

[19] Masood M, Newton T, Bakri NN, Khalid T, Masood Y (2017). The relationship between oral health and oral health-related quality of life among elderly people in the United Kingdom. J of Dentistry.

[20] Hugo FN, Hilgert JB, de Sousa Mda L, Cury JA (2009). Oral status and its association with general quality of life in older independent-living south-Brazilians. Community Dent Oral Epidemiol.

[21] Akifusa S, Soh I, Ansai T, Hamasaki T, Takata Y, Yohida A (2005). Relationship of number of remaining teeth to health-related quality of life in community-dwelling elderly. Gerodontology.

[22] Fontanive V, Abegg C, Tsakos G, Oliveira M (2013). The association between clinical oral health and general quality of life: a population based study of individuals aged 50–74 in southern Brazil. Community Dent Oral Epidemiol.

[23] Agarwal DP (2002). Cardioprotective effects of light-moderate consumption of alcohol: a review of putative mechanisms. Alcohol.

[24] Oh JY, Yang YJ, Kim BS, Kang JH (2007). Validity and reliability of Korean version of international physical activity questionnaire (IPAQ) short form. J Korean Acad Fam Med.

[25] Kim SW, Han K, Kim SY, Park CK, Rhee CK, Yoon HK (2016). The relationship between the number of natural teeth and airflow obstruction: a cross-sectional study using data from the Korean National Health and nutrition examination survey. Int. J. Chron. Obstruct. Pulmon. Dis..

[26] Laguna L, Sarkar A, Artigas G, Chen J (2015). A quantitative assessment of the eating capability in the elderly individuals. Physiol Behav.

[27] Kim SH, Ahn J, Ock M (2016). The EQ-5D-5L valuation study in Korea. Qual Life Res.

[28] Kwon KM, Lee JS, Jeon NE, Kim YH (2017). Factors associated with health-related quality of life in Koreans aged over 50 years: the fourth and fifth Korea National Health and nutrition examination survey. Health Qual Life Outcomes.

[29] Chun YH, Kim HR, Han K, Park Y-G, Song HJ, Na K-S (2013). Total cholesterol and lipoprotein composition are associated with dry eye disease in Korean women. Lipids Health Dis.

[30] Lee HO, Kim J (2008). Effects of elder' oral health beliefs and oral health behaviors on their quality of life. J Dent Hyg Sci.

[31] Han JH, Yom YH (2012). Effects of eating habits, activities of daily living and health behaviors on oral health related-quality of life in elderly persons. J Korean Acad Fundam Nurs.

[32] Brazier J, Roberts J, Deverill M (2002). The estimation of a preference-based measure of health from the SF-36. J Health Econ.

[33] Kaplan RM, Anderson JP (1998). A general health policy model: update and application. Health Serv Res.

[34] Torrance GW, Feeny DH, Furlong WJ, Barr RD, Zhang Y, Wang Q (1996). Multiattribute utility function for a comprehensive health status classification system: health utilities index mark 2. Med Care.

[35] Feeny D, Furlong W, Torrance GW, Goldsmith CH, Zhu Z, DePauw S (2002). Multiattribute and single-attribute utility function the health utility index mark 3 system. Med Care.

[36] WHOQoL Group (1995). The World Health Organization quality of life assessment (WHOQoL): position paper from the World Health Organization. Soc Sci Med.

[37] Hoi LV, Chuc NT, Lindholm L (2010). Health-related quality of life, and its determinants, among older people in rural Vietnam. BMC Public Health.

[38] Lang HC, Chuang L, Shun SC, Hsieh CL, Lan CF (2010). Validation of EQ-5D in patients with cervical cancer in Taiwan. Support Care Cancer.

[39] Lee Y-H, Choi J-S, Rhee J-A, Kim J-H (2009). A study on the application of the Korean valuation weights for EuroQoL-5 dimension. Korean J Health Educ Promot.

[40] Brennan DS, Teusner DN (2015). Oral health impacts on self-rated general and oral health in a cross-sectional study of working age adults. Community Dent Oral Epidemiol.

[41] Brennan DS, Singh KA (2012). Dietary, self-reported oral health and sociodemographic predictors of general health status among older adults. J Nutr.

[42] Brennan DS, Singh KA (2011). General health and oral health self-ratings, and impact of oral problems among older adults. Eur J Oral Sci.

[43] Back JU (2012). The effect of oral health on total health and quality of life between Korean and Japanese. KPHR.

[44] Kandelman D, Petersen PE, Ueda H (2008). Oral health, general health, and quality of life in older people. Spec Care Dent.

[45] Cushing AM, Sheiham A, Maizels J (1986). Developing sociodental indicators-the social impact of dental disease. Community Dent Health.

[46] Gil-Montoya J, De Mello A, Barrios R, Ganzalez-Moles M, Bravo M (2015). Oral health in the elderly patient and its impact on general well-being: a nonsystematic review. Clin Interv Aging.

[47] Locker D, Slade G (1994). Association between clinical and subjective indicators of oral health status in an older adult population. Gerodontology.

[48] Miura H, Yamasaki K, Morizaki N, Moriya S, Sumi Y (2010). Factors influencing oral health-related quality of life (OHRQoL) among the frail elderly residing in the community with their family. Arch Gerontol Geriatr.

[49] Catović A, Bergman V, Catić A (2003). Qualitative evaluation of elderly home residents' fixed and removable prostheses in relation to the ADL index. J Dent.

[50] Naito M, Kato T, Fujii W, Ozeki M, Yokoyama M, Hamajima N (2010). Effects of dental treatment on the quality of life and activities of daily living in institutionalized elderly in Japan. Arch Gerontol Geriatr.

[51] Centers for Disease Control & Prevention Disease Prevention Center Chronic Disease Control Researcher, Seoul National University School of Dentistry Preventive and Public Health Dentistry. The association between periodontitis and systemic disease. http://cdc.go.kr. Accessed 25 Apr 2017.

[52] Sandberg GE, Sandberg HE, Wikblad KF (2001). A controlled study of oral self-care and self-perceived oral health in type 2 diabetic patients. Acta Odontol.

[53] Loesche WJ (1994). Periodontal disease as a risk factor for heart disease. Compend Contin Educ Dent.

[54] Buhlin K, Gustasson A, Hakansson J (2002). Oral health and cardiovascular disease in Sweden. J Clin Periodontal.

[55] Makhija SK, Gilbert GH, Clay OJ, Matthews JC, Sawyer P, Allman RM (2011). Oral health–related quality of life and life-space mobility in community-dwelling older adults. J Am Geriatr Soc.

[56] Kwon HG. The baseline study of the denture treatment program for low socio-economic elderly people. The Ministry of Health and Welfare; 2002. http://www.ndsl.kr/ndsl/commons/util/ndslOriginalView.do?dbt=TRKO&cn=TRKO201400020409&rn=&url=&pageCode=PG18. Accessed 28 Apr 2017.

[57] Cassolato SF, Tumbull RS (2003). Xerostomia: clinical aspects and treatment. Gerodontology.

[58] Knapp A (1989). Nutrition and oral health in the elderly. Dent Clin N Am.

[59] Heydecke G, Locker D, Awad MA, Lund JP, Feine JS (2003). Oral and general health-related quality of life with conventional and implant dentures. Community Dent Oral Epidemiol.

[60] Sonoyama W, Kuboki T, Okamoto S, Suzuki H, Arakawa H, Kanyama M (2002). Quality of life assessment in patients with implant-supported and resin-bonded fixed prosthesis for bounded edentulous spaces. Clin Oral Implant Res.

[61] Heydecke G, Tedesco LA, Kowalski C, Inglehart MR (2004). Complete dentures and oral health-related quality of life-do coping styles matter?. Community Dent Oral Epidemiol.

